# The ability to regulate voltage-gated K^+^-permeable channels in the mature root epidermis is essential for waterlogging tolerance in barley

**DOI:** 10.1093/jxb/erx429

**Published:** 2017-12-28

**Authors:** Muhammad Bilal Gill, Fanrong Zeng, Lana Shabala, Jennifer Böhm, Guoping Zhang, Meixue Zhou, Sergey Shabala

**Affiliations:** 1 Department of Agronomy, Zhejiang University, Hangzhou, China; 2 School of Land and Food, University of Tasmania, Hobart, Tasmania, Australia

**Keywords:** GORK, H^+^-ATPase, H^+^-PPase, hypoxia, ionic homeostasis, potassium, signalling, viability staining, waterlogging

## Abstract

Oxygen depletion under waterlogged conditions results in a compromised operation of H^+^-ATPase, with strong implications for membrane potential maintenance, cytosolic pH homeostasis, and transport of all nutrients across membranes. The above effects, however, are highly tissue specific and time dependent, and the causal link between hypoxia-induced changes to the cell’s ionome and plant adaptive responses to hypoxia is not well established. This work aimed to fill this gap and investigate the effects of oxygen deprivation on K^+^ signalling and homeostasis in plants, and potential roles of GORK (depolarization-activated outward-rectifying potassium) channels in adaptation to oxygen-deprived conditions in barley. A significant K^+^ loss was observed in roots exposed to hypoxic conditions; this loss correlated with the cell’s viability. Stress-induced K^+^ loss was stronger in the root apex immediately after stress onset, but became more pronounced in the root base as the stress progressed. The amount of K^+^ in shoots of plants grown in waterlogged soil correlated strongly with K^+^ flux under hypoxia measured in laboratory experiments. Hypoxia induced membrane depolarization; the severity of this depolarization was less pronounced in the tolerant group of cultivars. The expression of *GORK* was down-regulated by 1.5-fold in mature root but it was up-regulated by 10-fold in the apex after 48 h hypoxia stress. Taken together, our results suggest that the GORK channel plays a central role in K^+^ retention and signalling under hypoxia stress, and measuring hypoxia-induced K^+^ fluxes from the mature root zone may be used as a physiological marker to select waterlogging-tolerant varieties in breeding programmes.

## Teaser

GORK channel-mediated K^+^ retention in roots is central to adaptation and signalling under hypoxia; this trait may be used as a physiological marker in waterlogging tolerance-oriented breeding programmes.

## Introduction

Waterlogging (WL) is a major environmental constraint limiting agricultural production worldwide and hampering 10% of the global land area (Setter and Waters, 2003). The estimated annual financial loss in agricultural production due to floods exceeds €60 billion (Dobrovičová *et al.*, 2015). Global climate changes are predicted to increase the rate and severity of flooding events in a large number of agricultural and urban areas of the world during this century (Arnell and Liv, 2001; Seneviratne *et al.*, 2012; Hirabayashi *et al.*, 2013). Under waterlogged conditions, the level of oxygen in the soil drops down rapidly from 230 nmol m^−3^ (well-drained soil) to 50 nmol m^−3^ (hypoxic) (Turner and Patrick, 1968) or may even result in a complete absence of oxygen (anoxia), due to high microbial activities (Ponnamperuma, 1984). Under these hypoxic and anoxic conditions, the resultant O_2_ deficiency and accumulation of CO_2_ in the root zone limit the root metabolism, aerobic respiration, and ATP synthesis, affecting the growth of shoots and roots (Gibbs and Greenway, 2003; Bailey-Serres and Colmer, 2014). In addition, hypoxia also limits the availability of required energy to fuel the H^+^-ATPase pumps and severely affects the transportation of ions which ultimately affect the growth and yield (Bailey-Serres and Voesenek, 2008; Elzenga and van Veen, 2010).

Potassium (K^+^) is the second most abundant mineral element in plant tissues. Although the availability of K^+^ differs from ~0.025 mM to 5 mM in different soils depending on the soil type and other environmental factors (Maathuis, 2009), cytosolic K^+^ concentrations in plants are sustained at a level of ~150 mM (Kronzucker *et al.*, 2008; Shabala *et al.*, 2016) to enable the essential role of K^+^ in activating and regulating nearly 70 different metabolic enzymes in plants (Dreyer and Uozumi, 2011). Potassium also plays an important role as a determinant of cell fate, with cytosolic K^+^ acting as a trigger of programmed cell death under a range of biotic and abiotic stress conditions (Shabala, 2009; Demidchik *et al.*, 2010). The plant’s ability to take up K^+^ is significantly reduced under O_2_-deficient conditions (Kreuzwieser and Gessler, 2010; Zhao *et al.*, 2012; Zeng *et al.*, 2014), but detrimental effects of WL on plants may be ameliorated by the exogenous application of K^+^ to soil or as a foliar spray (Ashraf *et al.*, 2011; Wang *et al.*, 2013).

Although the important role of maintaining intracellular K^+^ homeostasis in plant adaptive responses to flooding stress has been repeatedly mentioned on many occasions (Pang *et al.*, 2004; Colmer and Greenway, 2010; Mugnai, 2011; Shabala *et al.*, 2016), some controversies were reported in the previously published studies. While a significant decline in the shoot K^+^ content under waterlogged conditions was reported in many species [e.g. eucalypts (Close and Davidson, 2003); *Hordeum marinum* (Malik *et al.*, 2008); wheat (Trought and Drew, 1980); lucerne (Smethurst *et al.*, 2005); soybean (Board, 2008)], results on roots are more controversial. The reported results range from a substantial decline [e.g. lucerne (Smethurst *et al.*, 2005); wheat (Buwalda *et al.*, 1988); cucumber (Alcántara *et al.*, 1991); cherrybark oak (Pezeshki *et al.*, 1999)] to no change or even an increase [e.g. corn (Ashraf and Rehman, 1999); baldcypress ( Pezeshki *et al.*, 1999); *Lotus tenuis* (Teakle *et al.*, 2010)] in root K^+^ content. In a study using two halophyte grasses (*Puccinellia ciliata* and *Thinopyrum ponticum*) differing in WL stress tolerance (Teakle *et al.* 2013), the WL-tolerant *P. ciliata* showed a significant uptake of K^+^ under oxygen-deprived conditions, while the more sensitive *T. poticum* showed a substantial K^+^ leakage when measured by the microelectrode ion flux estimation (MIFE) system. The possible explanations for the above controversies may lay in the fact that both transport and regulation of K^+^ homeostasis under O_2_-limited conditions should be put into the context of the root tissue and genotypic specificity. Also, even though the link between the root’s ability to retain K^+^ and the WL stress tolerance has been reported (Pang *et al.*, 2006; Zeng *et al.*, 2014), the molecular mechanisms underlying this phenomenon remain unclear. K^+^ transport across the plasma membrane (PM) is mediated by a very large number (75 in Arabidopsis) of transport systems, and which of them plays a major role in hypoxia response remains to be elucidated.

The PM H^+^-ATPases generate the proton motive force (Sondergaard *et al.*, 2004) and thus are central to the maintenance of membrane potential (MP) and nutrient uptake by roots. Oxygen deficiency rapidly depolarizes the PM potential of WL-sensitive species of higher plants (Buwalda *et al.*, 1988; Teakle *et al.*, 2013). As a result, the channel-mediated uptake of many essential cations (e.g. K^+^, Mg^2+^, and NH_4_^+^) is reduced or becomes thermodynamically not feasible. Also, uptake of most of the essential anions (e.g. NO_3_^−^ or SO_4_^2−^ and PO_4_^2−^) is reduced, as a consequence of a reduced driving force for H^+^-coupled symport systems (Kreuzwieser and Gessler, 2010; Wang *et al.*, 2013). Many biotic and abiotic stresses induce K^+^ leakage from plant tissues; in most cases, this K^+^ efflux is mediated by depolarization-activated outwardly-rectifying K^+^ (GORK) channels (Demidchik *et al.*, 2014; Shabala and Pottosin, 2014). The GORK channel displayed high expression in guard cells, roots (epidermal cells, cortex, and root hairs), and cells of the vascular tissue (Gambale and Uozumi, 2006; Ward *et al.*, 2009). The expression and activity of GORK channels were both substantially affected under drought, salt, and cold stress conditions (Becker *et al.*, 2003), and these channels were named as a major pathway for stress-induced K^+^ leakage in many plant species exposed to salt stress (Shabala and Cuin, 2008; Jayakannan *et al.*, 2013). A pharmacological study suggested that the hypoxia-induced K^+^ leakage may also be mediated by KOR channels, as K^+^ efflux in barley roots was strongly inhibited (Pang *et al.*, 2006) by the application of tetraethylammonium, a known blocker of the voltage-gated Shaker-type K^+^ channel to which GORK belongs. In a recent study from our laboratory, we have shown (Wang *et al.* 2016) that the expression of *GORK* was down-regulated in hypoxic root cells of wild-type Arabidopsis. In addition, the mutant *gork1-1* lacking functional GORK channels showed significantly higher K^+^ accumulation in both elongation and mature zones under hypoxia stress, compared with the wild type. After 3 d of hypoxia stress, *gork1-1* showed a significantly smaller K^+^ efflux than the wild type in the elongation zone, and retained an influx in the mature zone while the wild type showed a K^+^ efflux (Wang *et al.*, 2016). The most likely explanation behind this tissue-specific K^+^ efflux would be the differential regulation ability of GORK channels in the elongation and mature root zones. Can the same conclusion be extrapolated from Arabidopsis to cereals? Are all root cells equally sensitive to hypoxia, or do some tissues have a higher demand for oxygen? Can hypoxia stress-induced K^+^ efflux be used as a physiological marker for WL stress tolerance? If yes, from where should it be measured?

The aim of this study was to fill the above gaps in our knowledge. To achieve this, we used contrasting barley cultivars to investigate tissue- and genotype-specific effects of hypoxia on K^+^ retention in roots. Also, the role of GORK channel in K^+^ release was investigated by measuring hypoxia-induced changes in root MP and correlating it with the extent of K^+^ efflux from the root. Our results showed that hypoxic conditions caused a significant loss of K^+^ in a time- and genotype-specific manner, affecting cell viability and K^+^ regulation, and led to the conclusion that the genotypic difference in WL stress tolerance in barley is conferred by the differential ability to regulate voltage-gated K^+^-permeable channels in the mature root epidermis.

## Materials and methods

### Plant material and growth conditions

Six barley (*Hordeum vulgare* L.) cultivars contrasting in WL tolerance were used in this study. Among these cultivars, CM72, TX9425, and Yerong are tolerant, and Gairdner Franklin, and Naso Nijo are sensitive to WL (Pang *et al.*, 2004; Zhou *et al.*, 2012). Seeds were acquired from China and the Australian Winter Cereal Collection Centre, and reproduced in the field, using Tasmanian Institute of Agriculture (TIA) facilities in Launceston. Seeds were surface sterilized with 10% commercial bleach (NaClO 42 g l^−1^; Pental Products, Shepparton, Australia), thoroughly rinsed with tap water (for at least 30 min), and then grown in wet paper rolls with basic salt medium (BSM) solution (0.5 mM KCl+0.1 mM CaCl_2_, pH 5.6) in the dark for 3 d at room temperature (25 ± 1 °C). Two treatments were used in electrophysiological experiments: (i) control (BSM, aerated); and (ii) hypoxia (BSM solution made with 0.2% agar and bubbled with N_2_ gas). For the treatment with agar, the stagnant solution was prepared by adding agar (Cat. No. LP0011, Oxoid, Hampshire, UK) to the BSM solution at a ratio of 0.2% (w/v) and boiled, then cooled down overnight at room temperature with magnetic stirring to prevent formation of lumps. The agar solutions were pre-bubbled with high purity N_2_ (Cat. No. 032G, BOC Gases, Hobart, Australia) for at least 1 h before being used in the experiment.

### Glasshouse experiments

For glasshouse experiments, seeds of the above six barley varieties were grown in 2 litre pots filled with sandy loam soil. The soil was collected from the University of Tasmania farm and mixed with essential macronutrients (in g l^–1^: 0.51 NH_4_NO_3_, 1.35 NaH_2_PO_4_, 0.48 K_2_SO_4_, 0.31 CaSO_4_, and 0.14 MgCl_2_). During the germination stage, plants were watered with tap water at field capacity. Germinated plants were then thinned to eight uniform and healthy plants in every pot. Plants were grown under controlled glasshouse conditions (with a day-length of 14 h; light/dark temperatures of 25/15 °C; and relative humidity of 65–75%) at the University of Tasmania (Hobart, Australia) in March–April 2014.

Treatments were imposed when plants were 10 d old. Two treatments were given: control (well aerated) and WL (submerged pots). For treatment with WL, pots were placed into large containers (four pots in each container). WL conditions were imposed by using half-strength Hoagland’s nutrient solution (Hoagland and Arnon, 1950). The water level of waterlogged treatment was kept 15 mm above the soil surface. Control (well-aerated) plants were irrigated on a daily basis with 150 ml of half-strength Hoagland’s nutrient solution. Plants were subjected to treatments for 4 weeks. Leaf chlorophyll content was measured from the centre of the fully expanded leaves using a SPAD meter (SPAD-502, Minolta, Japan). Before harvesting for biomass, the number of chlorotic, necrotic, and total leaves from each plant were counted. The relative amount of chlorotic and/or necrotic leaves was then calculated according to the equation: ratio of chlorotic (or necrotic) leaves=no. of chlorotic (necrotic) leaves/no. of total leaves. Ten replicates were randomly taken for each treatment×variety combination. Fresh weight of shoots was recorded after harvesting, and the dry weight was measured after drying in a UniTherm Drier (Birmingham, UK) for 2 d at 65 °C. Four replicates each consisting of four plants were used for each treatment.

### Viability staining

For viability staining, surface-sterilized seeds of different barley cultivars were grown in aerated hydroponic BSM solution in the dark for 2 d at 25 ± 2 °C. On the next day, seedlings were transferred into a 14 h/10 h light/dark regime for 1 d. The root area of plants was protected from direct light by using black containers. Two treatments, control and hypoxia (0.2% agar), were imposed and lasted for 48 h. The cell viability was measured by using a fluorescein diacetate (FDA)–propidium iodide (PI) double staining method, principally as described by Koyama *et al.* (1995). The settings of the camera for all experiments were kept constant during image acquisition. The green fluorescence signal intensity (a measure of the cell viability) was quantified by using ImageJ software (version 1.48, Java, 64 bit).

### Tissue elemental analysis

For tissue elemental analysis, plants were harvested after 4 weeks of treatment. Plant shoots were washed three times with distilled water and blotted to remove excess water. Plants were kept at 80 °C for 48 h in an oven and then ground into a powder. Plant samples (0.2 g) were digested with a mixture of 5 ml of HNO_3_+1 ml of HClO_4_ in an infrared digestion furnace (PEIOU-SKD-20N, China). The resultant solutions were diluted to 25 ml using 2% HNO_3_ and filtered with a 0.45 µm filter paper. The concentration of K^+^ in the filtrate was determined using inductively coupled plasma–atomic emission spectrometry (IRIS/AP optical emission spectrometer) following a standard procedure.

### MIFE ion flux measurements

Net fluxes of K^+^ and H^+^ were measured from 60 ± 10 mm long roots by using a non-invasive ion flux measurement (MIFE) technique (University of Tasmania, Hobart, Australia). The theory of MIFE measurements and other details of calibration and fabrication related to ion-selective microelectrode are given in our previous publications (Shabala *et al.*, 2006, 2010). In brief, borosilicate microelectrodes were filled with appropriate backfilling solution. Electrode tips were then filled with an appropriate liquid ion exchanger (LIX) (Fluka catalogue no. 60031 for K^+^ and 95297 for H^+^). After calibration in a set of pH and different K^+^ standards, the electrodes were mounted on a 3D micromanipulator. Electrodes were then positioned 40 µm from the root surface and placed on the same plane 2–3 µm from each other. During the measurement of fluxes, microelectrodes were moving between two positions: 40 µm (close to the root epidermis) and away (90 µm) in a 10 s cycle square-wave manner. The potential difference between these two points was recorded by the MIFE CHART software and converted into electrochemical potential difference by using cylindrical diffusion geometry (Newman, 2001).

Prior to measurement, a 3-day-old seedling was taken from a paper roll and immobilized horizontally by the Plexiglass partitions 2 mm above the surface of the chamber (see Supplementary Fig. S1 at *JXB* online). The measuring chamber was filled with hypoxia solution, with the coleoptile being above the surface of the solution. Seedlings for control were well aerated during the period of treatment, whereas hypoxia- (0.2% agar) treated seedlings were kept in stagnant conditions for different times, ranging from 2 h to 48 h. The seedlings were then placed in a Faraday cage for MIFE measurements. Net ion fluxes were measured for 10 min; the steady-state fluxes were achieved by averaging the values of the last 5 min. For each treatment, net ion fluxes were measured from intact roots of at least 6–8 individual seedlings.

In some experiments, excised roots were used to eliminate the possibility of oxygen supply through coleoptiles and to restrict internal oxygen movement. For this purpose, a 3-day-old seedling was taken from the paper roll and the coleoptile was removed gently with a scalpel blade. The excised roots were placed in a Faraday cage for MIFE measurements after giving different treatments as described in the previous paragraph. For each treatment, net ion fluxes were measured from 6–8 individual roots with excised coleoptiles.

### Membrane potential measurements

MP was measured from epidermal cells of intact barley roots. The MP measurements were performed as described previously (Cuin and Shabala, 2005; Bose *et al.*, 2010). Briefly, conventional microelectrodes (Harvard Apparatus) were filled with 1 M KCl and connected to the MIFE electrometer via an Ag/AgCl half-cell. During MP measurement, the microelectrode with a tip diameter of 0.5 µm was impaled into the epidermal cells of the mature root zone with a manually operated 3D micromanipulator (MHW-4, Narishige, Tokyo, Japan). MP values were recorded by the MIFE CHART software for at least 2 min after stabilization (Newman, 2001). For MP measurements, the barley seedlings were mounted in the vertical chambers and treated with a hypoxic solution as described for MIFE experiments. For each treatment, MP values were measured from roots of 5–6 individual seedlings. At least four different cells were measured for each seedling. MP measurements were made either before (time zero) or after 48 h of treatment.

### Pharmacology

Roots pre-treated with hypoxia stress (0.2% agar) for 24 h and control roots were used for pharmacological experiments. Net ion fluxes were measured for 5 min in both control and hypoxia-treated roots, then 0.5 mM orthovanadate (a potent inhibitor of the H^+^-ATPase pump) was applied and steady-state fluxes were recorded for another 10 min. For each treatment, net ion fluxes were measured from roots of at least 6–8 individual seedlings.

### Quantitative real-time PCR

RNA from the root apices (4–5 mm long) from both mature and elongation zones of barley were isolated using the ISOLATE II RNA Plant Kit (Bioline) and purified by the ISOLATE II micro clean-up kit (Bioline) according to the manufacturers’ protocols. A 8000 ng aliquot of total RNA was reverse transcribed using a Sensi FAST cDNA Synthesis Kit (Bioline) in a total volume of 20 µl. RNA concentration was monitored witha Nano Drop 8000 UV-Vis Spectrophotometer (Thermo Scientific). Quantitative real-time PCRs (qRT-PCRs) were performed in a Roto-Gene 3000A (Corbett Research) with the Quanti Nova SYBR Green PCR Kit (Qiagen) in a 20 µl volume containing 2 µl of 5-fold diluted cDNA and 0.7 nM primer mix (1:1 mix of forward and reverse primers). After a hot start (5 min at 95 °C), a two-step PCR program was applied: 45 times for 10 s at 95 °C, and 30 s at 60 °C, followed by a dissociation curve by increasing the temperature every 1 °C from 55 °C to 99 °C (with a 5 s hold at each temperature). Primers (Gene Works) are listed in Supplementary Table S1. All quantifications were normalized to 10 000 molecules of the housekeeping gene *HvGAPDH* and each transcript was quantified using individual standards. For each gene, the quantification in the mature and elongation zones of control conditions was also normalized to 1 (corresponding to 100%). Three to five technical and biological replicates were performed for each experiment and treatment.

### Statistical analysis

Statistical analysis was performed by the statistical package IBM SPSS Statistics 20 (IBM, New York, NY, USA). All data in the table and figures are given as means ±SE. The significant difference between means was evaluated by Duncan’s multiple range test.

## Results

### Genotypic variation in plant growth under waterlogging

**Fig. 1.**
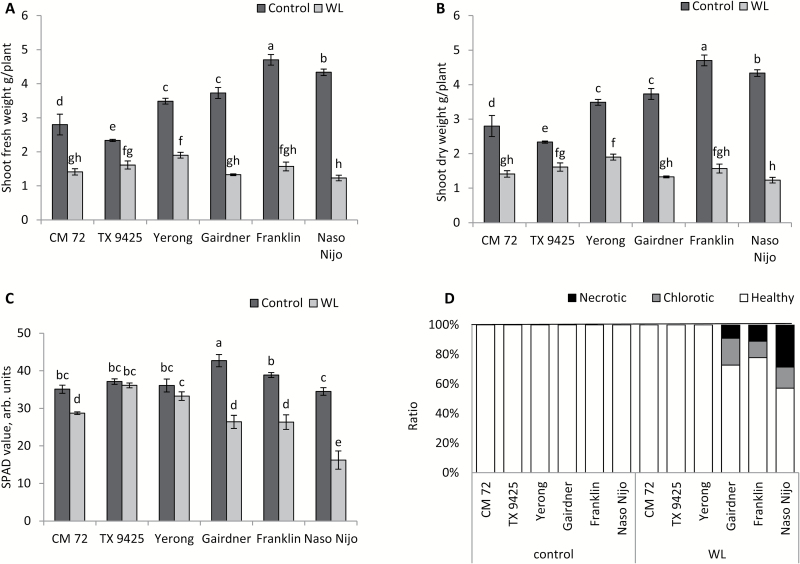
Effects of waterlogging (WL) on agronomical and physiological characteristics of six contrasting barley cultivars. (A) Shoot fresh weight; (B) shoot dry weight; (C) chlorophyll content (SPAD values); and (D) the ratio of chlorotic and necrotic leaves (calculated as the number of chlorotic or necrotic leaves divided by the total number of leaves). Values are means ±SE (*n*=4). Different lower case letters indicate a significant difference at *P*≤0.05 according to Duncan’s multiple range tests.

A glasshouse experiment was undertaken to assess the genotypic variation in a range of barley genotypes under WL stress. Four weeks of WL stress affected the growth of all cultivars, but to a different extent. The most severe effect of WL on plant growth was observed in cultivars Gairdner, Franklin, and Naso Nijo (Fig. 1). These varieties were therefore termed WL sensitive, in contrast to the other three termed WL tolerant. When these cultivars were grown in sandy loam soil for 4 weeks, the average shoot fresh weights of tolerant and sensitive cultivars were reduced by 42% and 68% relative to the control, respectively; for shoot dry weight, these values were 32% and 61% (Fig. 1A, B). The chlorophyll content in the tolerant and sensitive group of cultivars was reduced by 9% and 41% as compared with the respective controls (Fig. 1C). The effect of WL treatment on chlorophyll content (SPAD value) was not significant (*P*<0.05) in tolerant cultivars TX9425 and Yerong (Fig. 1C). The results showed that WL treatment induced the chlorosis and necrosis of leaves, and the tolerant cultivars performed much better as compared with the sensitive cultivars (Fig. 1D). There were no chlorotic and necrotic signs on leaves of WL-tolerant cultivars, while in sensitive cultivars 21% chlorotic and 26% necrotic leaves were observed on average under WL treatment (Fig. 1D).

### Hypoxia-grown barley cultivars differed in cell viability and whole-plant K^+^ content

**Fig. 2.**
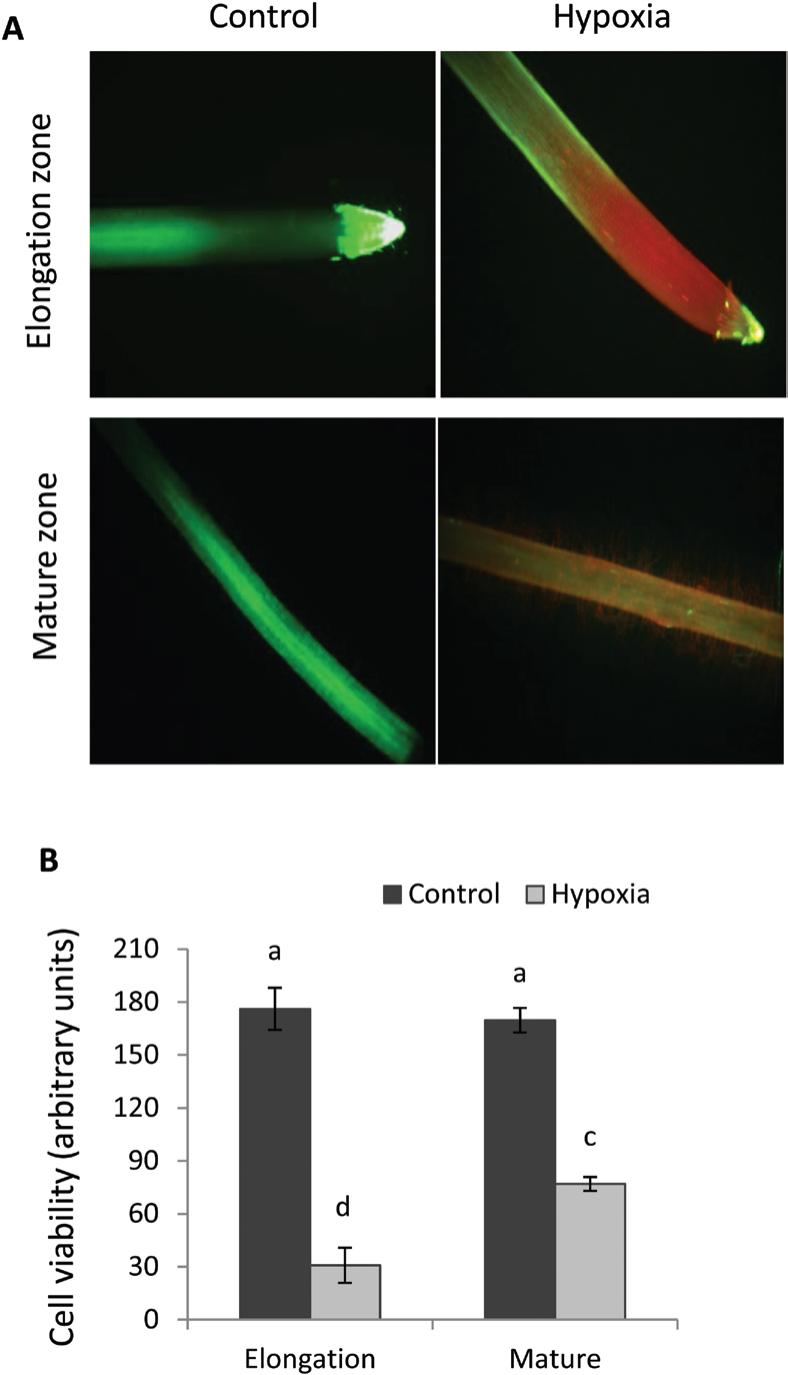
Effects of hypoxia (N_2_-bubbled 0.2% agar) on root viability of a waterlogging-sensitive barley cultivar (Gairdner). (A) Barley roots were stained with fluorescein diacetate–propidium iodide (FDA–PI). Control and 48 h hypoxia-treated barley roots stained with FDA (5 µg ml^–1^ for 5 min) and PI (3 µg ml^–1^ for 10 min) for fluorescence imaging. Viable cells displayed green fluorescence due to FDA, and non-viable cells displayed red fluorescence due to PI. One (of six) typical image is shown for each zone (elongation zone, ~3 mm from the root tip; mature zone, ~5–10 mm from the shoot base). (B) Quantification of the cell viability from mature and elongation zones of barley roots. The intensity of the green fluorescence signal (a measure of cell viability) was quantified by Image J software. Data are the mean ±SE (*n*=6–8 individual plants). Scale bar=0.5 mm. Different lower case letters indicate a significant difference at *P*≤0.05 according to Duncan’s multiple range tests.

**Fig. 3.**
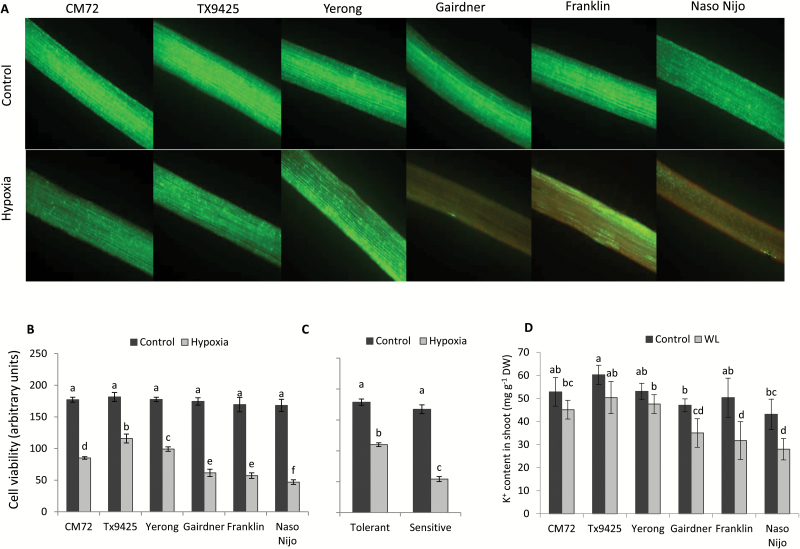
(A) Genotypic variation in effects of hypoxia (N_2_-bubbled 0.2% agar) on root viability of six barley cultivars differening in waterlogging (WL) stress tolerance. Barley roots were stained with fluorescein diacetate–propidium iodide (FDA–PI). Viable cells displayed green fluorescence due to FDA, and non-viable cells displayed red fluorescence due to PI. One (of six) typical image is shown for each cultivar in the mature zone, ~5–10 mm from the shoot base. The intensity of the green fluorescence signal (a measure of cell viability) was quantified by Image J software. Scale bar=0.5 mm. (B) Quantification of the cell viability from the mature root (5–10 mm from the shoot base). (C) Mean pooled cell viability quantification values for three tolerant and three sensitive varieties measured as above. (D) Effects of 4 weeks WL stress on K^+^ content in shoots of six contrasting barley cultivars grown in sandy loam soil (mg g^–1^ DW). Data are mean ± SE (*n*=6–8 individual plants). Different lower case letters indicate a significant difference at *P*≤0.05 according to Duncan’s multiple range tests.

We next used the viability staining technique to comprehend tissue-specific effects of hypoxia on cell metabolism (Fig. 2). A significant (*P*<0.05) loss of viability was found in both the elongation and mature zone after hypoxia (Fig. 2B). The apical cells were more severely damaged as compared with the mature zone (as indicated by the deep red colour in Fig. 2A). However, a very strong heterogeneity of the fluorescent signal from the apex (Fig. 2A) has questioned the suitability of this zone as a physiological marker in screening programmes. In this context, the signal from the mature zone was more uniform and thus more suitable for screening purposes. Thus, we have used this region for comparing effects of hypoxia on root cell viability among six contrasting barley cultivars (Fig. 3A). There was no sign of any viability loss in tolerant cultivars in the mature zone, but clear signs of the viability loss were observed in sensitive cultivars when compared with appropriate controls (Fig. 3A). The above visual observations were then quantified, revealing a statistically significant (at *P*<0.05) difference between two groups (Fig. 3B, C). In sensitive cultivars, the loss of viability was almost 1.5- to 2-fold greater than in tolerant cultivars (Fig. 3C). The loss of cell viability in hypoxia-treated roots mirrored the changes imposed by WL stress in shoot K^+^ content of glasshouse-grown barley cultivars (Fig. 3D). Here, sensitive cultivars lost 1.5- to 2-fold more K^+^ in the shoot, as compared with tolerant cultivars.

### Long-term hypoxic treatment is more detrimental to root ion fluxes

**Fig. 4.**
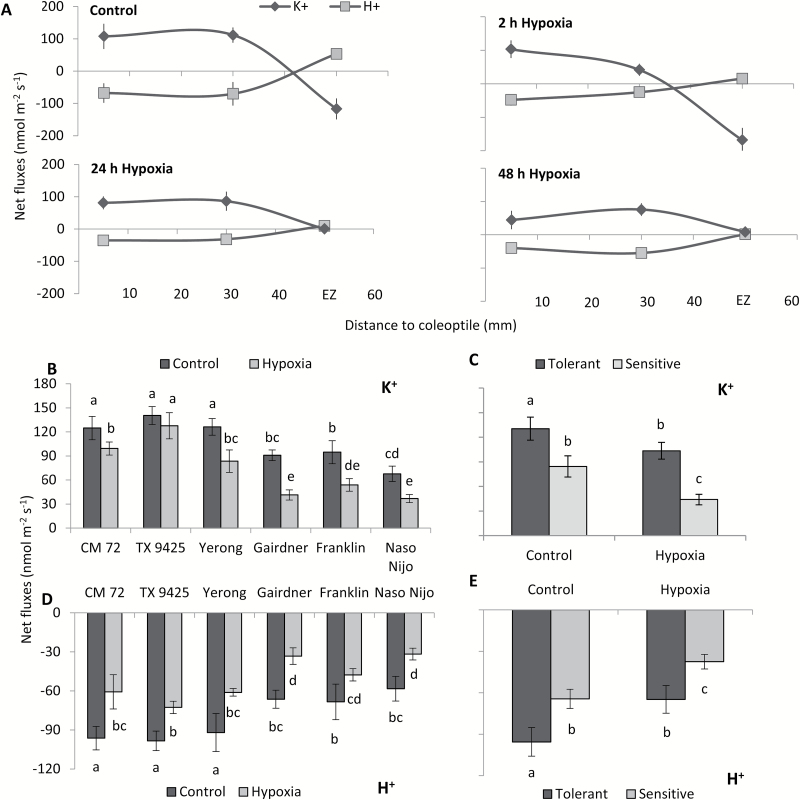
Effects of hypoxia (N_2_-bubbled 0.2% agar) on net K^+^ and H^+^ fluxes measured from barley root epidermis of intact seedlings. (A) Mean fluxes were measured from the intact roots of a waterlogging-sensitive barley cultivar (Gairdner) in control and after 2, 24, and 48 h of hypoxia treatment from different zones along the root axis. EZ indicates the elongation zone figure. (B) Net K^+^ flux measured after 48 h of hypoxia treatment in the mature root zone (5 mm from the root base) of six barley cultivars contrasting in waterlogging tolerance. (C) Mean pooled K^+^ values for three tolerant and three sensitive varieties measured as above. (D) Net H^+^ flux measured after 48 h of hypoxia treatment in the mature root zone (5 mm from root base) of six barley cultivars contrasting in waterlogging tolerance. (E) Mean pooled H^+^ values for three tolerant and three sensitive varieties measured as above. Data are the mean ±SE (*n*=6–8 individual plants). Different lower case letters indicate a significant difference at *P*≤0.05 according to Duncan’s multiple range tests.

Net K^+^ and H^+^ fluxes were measured from intact barley roots to examine the effects of hypoxia on the ion flux profile along the root axis. Different treatment durations were used, ranging between 2 h and 48 h. A significant uptake of K^+^ in the mature zone and a strong efflux in the elongation zone were observed under control conditions (Fig. 4A). Hypoxic treatment for 2 h increased net K^+^ efflux in the elongation zone; no significant change in net K^+^ flux was observed in the mature zone (Fig. 4A) at this time point. As hypoxia progressed, both K^+^ uptake (in the mature zone) and leakage (in the apex) were gradually reduced (24 h and 48 h hypoxic treatments). In the mature zone, no significant change was found for net H^+^ efflux, while a considerable reduction of H^+^ influx was observed in the elongation zone of intact roots after 2 h of hypoxia treatment (Fig. 4A). Steady-state H^+^ flux gradually decreased in both zones as the hypoxia treatment progressed.

A series of experiments were further conducted to verify the genotypic specificity of the detrimental effects of hypoxia on H^+^ and K^+^ fluxes (Fig. 4B–D) to see if hypoxia-induced ion fluxes could be used as a proxy for WL stress tolerance. The above six barley cultivars were used to compare ion fluxes under hypoxic conditions (48 h treatment). Measurements were conducted in the mature zone, 5 mm from the shoot base (Fig. 3). Hypoxia reduced the rate of H^+^ pumping and resulted in a significant decline in K^+^ uptake after 48 h of hypoxia (Fig. 4B, D). Tolerant cultivars showed significantly better abilities for H^+^ pumping and showed less decrease in K^+^ uptake than the sensitive cultivars under both control and hypoxic conditions (Fig. 4C, E), with tolerant cultivars showing 30% less K^+^ leakage (Fig. 4C) and 40% more H^+^ pumping (Fig. 4E) as compared with sensitive cultivars. The observed decrease in shoot K^+^ content showed a strong (*R*^2^=0.85) positive correlation with net K^+^ efflux from stressed roots measured in MIFE experiments (Supplementary Fig. S2).

### Disruption of internal O_2_ transport reduces ion fluxes under hypoxic conditions

**Fig. 5.**
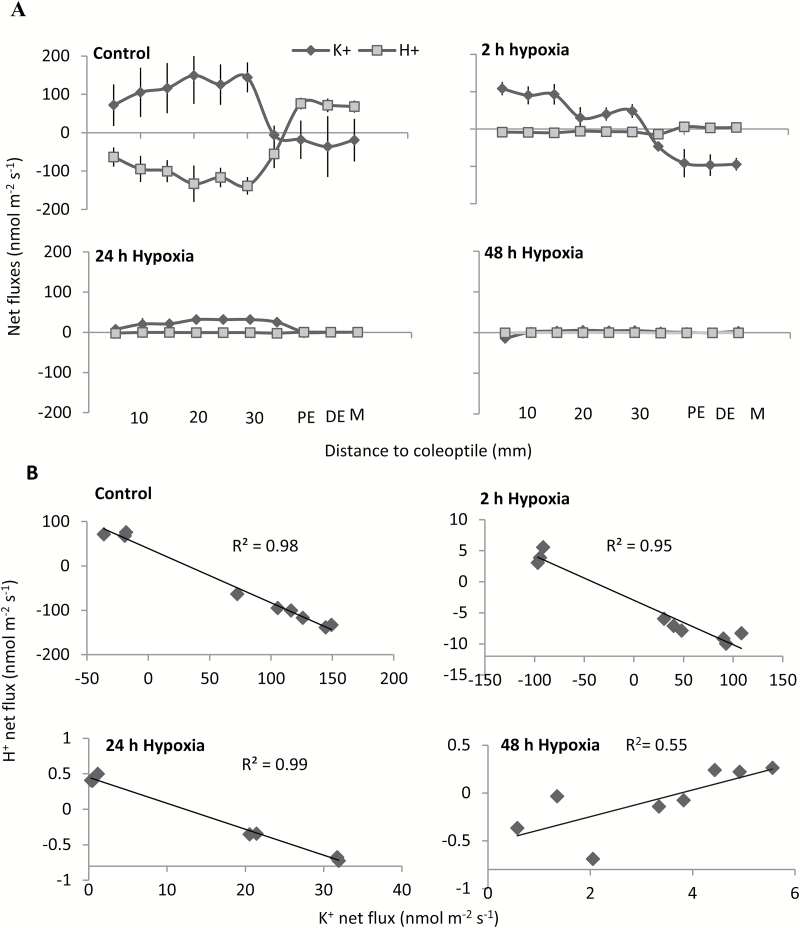
Net K^+^ and H^+^ flux response to hypoxia (N_2_-bubbled 0.2% agar) measured from excised roots of a waterlogging-sensitive cultivar (Gairdner). (A) Mean fluxes were measured along the barley root axis in control and after 2, 24, and 48 h of hypoxia treatment from different zones. P, pre-elongation; D, distal elongation; M, meristem. (B) Correlation between net K^+^ and H^+^ fluxes in control and hypoxia-treated roots measured at different time points. Data are the mean ±SE (*n*=6–8 individual plants).

In the above experiments, measurements were conducted on intact seedlings, with their coleoptiles being in the air and thus operating as a ‘snorkel’ supplying oxygen to root tissues even in the absence of oxygen in the growth medium (Zeng *et al.*, 2014). Thus, as a next step, we have investigated the effect of hypoxia on ion fluxes in plants where internal oxygen supply was prevented by excising coleoptiles. Net ion fluxes were measured from 10 different positions along the root axis (Fig. 5). Strong net H^+^ efflux and K^+^ uptake were measured from the mature zone of excised roots under control conditions (Fig. 5A). In contrast to the mature zone, the elongation zone showed an influx of H^+^ and an efflux of K^+^ (positions PE, pre-elongation; DE, distal elongation; and M, mature zone in Fig. 5A). The onset of hypoxia resulted in a reduced K^+^ uptake in the mature zone and further increased K^+^ leak from the root apex after 2 h of hypoxia treatment (Fig. 5B). This K^+^ loss in the apex was found to be transient and stopped after 24 h (Fig. 5A). In the mature zone, net K^+^ uptake was reduced to zero or even turned into net efflux after 48 h (Fig. 5A). The steady-state H^+^ flux was also reduced considerably after 2 h of hypoxia treatment in both zones as compared with controls (Fig. 4A). A significant reduction in H^+^ flux was measured as hypoxia progressed in both mature and elongation root zones after long-term hypoxic treatments (24 h and 48 h).

To understand the nature of transport systems mediating the observed effects of hypoxia on K^+^ fluxes in barley roots, a correlation analysis between K^+^ and H^+^ fluxes was conducted. A strong correlation between H^+^ and K^+^ fluxes existed at each time point (Fig. 5B), suggesting that the measured K^+^ fluxes were mediated by some voltage-gated transport system. These results further confirmed our previous finding (Zeng *et al.*, 2014) that the internal oxygen transportation played an important role in plant survival and performance under oxygen-limited conditions.

**Fig. 6.**
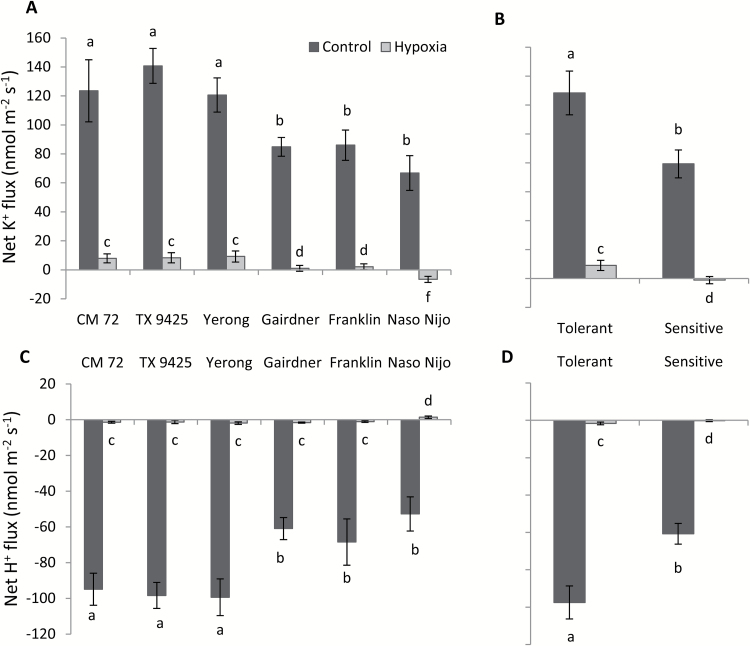
Effects of hypoxia (N_2_-bubbled 0.2% agar) on net K^+^ and H^+^ fluxes measured from excised roots of six barley cultivars contrasting in waterlogging stress tolerance. (A) Steady-state mean K^+^ fluxes (averaged over 5 min) measured after 48 h from the mature root zone (5 mm from the shoot base) of six contrasting cultivars from plants with excised coleoptiles. (B) Mean pooled K^+^ values for three tolerant and three sensitive varieties measured as above. (C) Steady-state mean H^+^ fluxes (averaged over 5 min) measured after 48 h from the mature root zone (5 mm from the shoot base) of six contrasting cultivars from plants with excised coleoptiles. (D) Mean pooled H^+^ values for three tolerant and three sensitive varieties measured as above. Data are the mean ±SE (*n*=6–8 individual plants). Different lower case letters indicate a significant difference at *P*≤0.05 according to Duncan’s multiple range tests.

Given the fact that preventing internal oxygen transportation by excising coleoptiles results in a stronger effect on ion profiles along the root axis, we next looked at the genotypic variation in this trait amongst barley genotypes, comparing effects of 48 h hypoxia treatment on K^+^ and H^+^ fluxes from the mature zone in six contrasting cultivars (Fig. 6). A trend similar to that observed for intact coleoptiles was found, with hypoxia reducing H^+^ pumping and K^+^ uptake. The detrimental effect of hypoxia was much stronger in sensitive cultivars as compared with tolerant cultivars (Fig. 6B, D). The observed difference was much more pronounced in plants with excised roots (Fig. 6) compared with intact plants (Fig. 4B), suggesting that the former are more suitable to be used in screening barley germplasm for WL stress tolerance by the MIFE technique.

### WL-tolerant cultivars maintain a more negative membrane potential

**Fig. 7.**
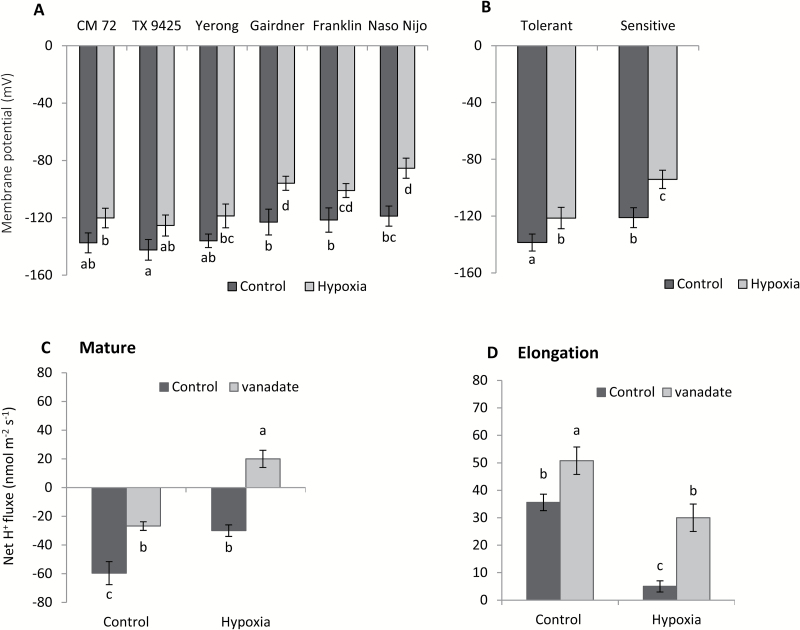
(A) Effects of hypoxia (N_2_-bubbled 0.2% agar) treatment after 48 h on the membrane potential of six contrasting barley cultivars measured from the mature root zone (5 mm from the shoot base). Data are the mean ±SE (*n*=18–30 measurements from at least five individual plants). (B) Mean pooled membrane potential values for three tolerant and three sensitive varieties measured as above. (C, D) Effect of vanadate (0.5 mM) on steady-state H^+^ fluxes measured from the mature and elongation regions of a barley waterlogging-sensitive cultivar (Gairdner) roots. Data are the mean ±SE (*n*=6 individual plants). Different lower case letters indicate a significant difference at *P*≤0.05 according to Duncan’s multiple range tests.

K^+^ efflux under stress conditions is largely mediated by the Shaker-like K^+^ channels (GORK in Arabidopsis; Shabala and Pottosin, 2014). Most Shaker-like channels are strongly voltage gated in nature. The GORK channel is not an exception and is classified as a depolarization-activated outward-rectifying K^+^ channel (reviewed in Anschütz *et al.*, 2014). Accordingly, we have measured the MP of epidermal root cells to check if the extent of hypoxia-induced K^+^ leak is correlated with stress-induced changes in MP. Our data showed that tolerant cultivars maintained more negative MP values as compared with sensitive cultivars, under both control and hypoxic conditions (Fig. 7A). When pooled together, MP values in the sensitive group were almost 10% less negative than in the tolerant group (Fig. 7B). MP depolarization was found to be consistent with the lower steady-state H^+^ flux in hypoxic conditions (Fig. 4D, E). This reduction in MP values under both conditions points to the involvement of the voltage-gated outward-rectifying K^+^ channels as the major path for the observed stress-induced K^+^ leak. This conclusion is further supported by pharmacological experiments using vanadate, a known blocker of H^+^-ATPase. In the mature zone, a significant reduction in H^+^ efflux was measured in control roots, while the efflux changed into an influx after vanadate application in the hypoxia-treated roots (Fig. 7D). Much higher H^+^ influx was measured in the elongation zone when vanadate was applied to both control and hypoxia-treated barley roots (Fig. 7C).

### Hypoxic-induced changes in relative expression of *GORK* and H^+^-pump-related genes

**Fig. 8.**
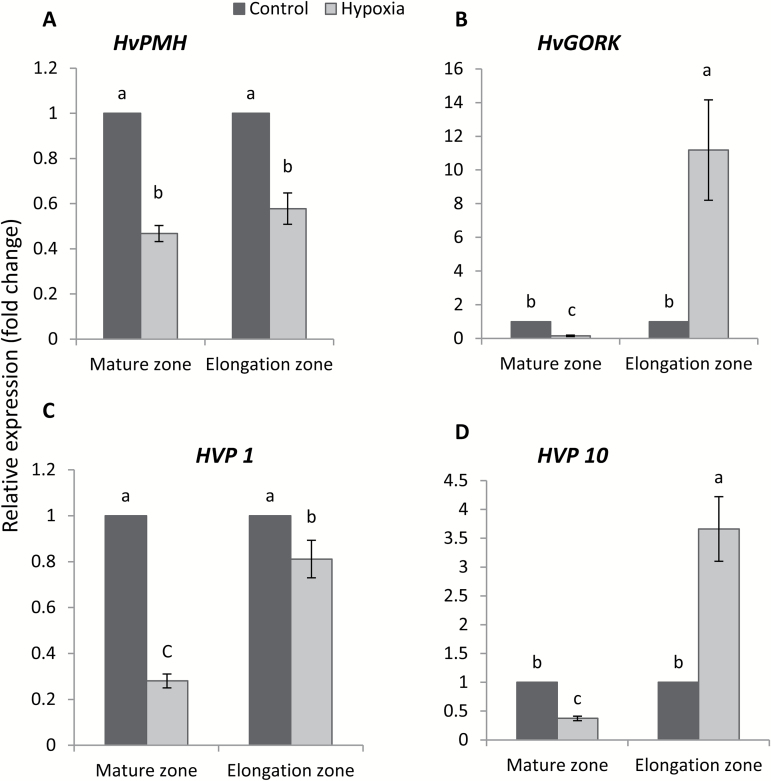
Effects of 48 h hypoxia (N_2_-bubbled 0.2% agar) treatment on relative expression of (A) H^+^-ATPase (*HvPMHA*), (B) KOR (*HvGORK*), (C) H^+^-HPPase (*HVP 1*), and (D) (*HVP 10*) in root tissues of a waterlogging-sensitive barley cultivar (Gairdner). Data are the mean ±SE (*n*=3–5 biological and technical replicates). Root apices (4–5 mm) long from both the elongation (elongation zone, ~3 mm from root tip) and mature zone (mature zone, ~5–10 mm from the shoot base) were taken for relative expression analysis. Different lower case letters indicate a significant difference at *P*≤0.05 according to Duncan’s multiple range tests.

We next compared the relative expression pattern of *GORK* genes and genes conferring PM and tonoplast H^+^-ATPase (HA) and H^+^-pyrophosphatase (VP) pumps, in two root zones. The relative transcript level of the H^+^-ATPase (*HvPMHA*) under hypoxia conditions showed a comparable pattern in the mature and elongation zone (Fig. 8A), reducing the expression by 53% and 42%, respectively. This reduction in the gene expression matches nicely the reported 40–60% reduction in net H^+^ flux measured by MIFE (Fig. 4A, D). By applying hypoxia, the relative expression of the K^+^ outward rectifier gene *HvGORK* decreased ~80% in the mature zone (Fig. 8B). In contrast, the *HvGORK* transcript level in the elongation zone showed a pronounced increase under hypoxia treatment, which resulted in a 10-fold higher *HvGORK* expression relative to the non-treated elongation zone. For both vacuolar H^+^-PPases (*HVP* genes), the relative expression in the mature zone decreased by >60% under hypoxia conditions (Fig. 8C, D), whereas in the elongation zone the hypoxia treatment resulted in a minor change of *HVP1* expression. In the elongation zone, the relative expression of *HVP10* was significantly higher (2.5-fold) under hypoxia treatment compared with the control condition.

## Discussion

### K^+^ retention confers cell viability and contributes to genotypic difference in WL tolerance in barley

Potassium is the most abundant inorganic cation in plant cells that plays a crucial role in numerous physiological processes including turgor maintenance, regulation of enzymatic activity, maintenance of membrane polarization, control of sugar and ion loading, and energy conservation processes (Dreyer and Uozumi, 2011; Shabala *et al.*, 2014). Potassium also plays an important role in plant adaptive responses to the hostile environment. The ability of plant tissue to retain K^+^ was positively correlated with salinity stress tolerance in a broad range of plant species including barley (Chen *et al.*, 2005), tomato (Heuer, 2003), wheat (Colmer *et al.*, 2006; Cuin *et al.*, 2008), lucerne (Smethurst *et al.*, 2008), rice (Ismail *et al.*, 2007), and poplar (Sun *et al.*, 2009). Early studies in our laboratory have also indicated that K^+^ retention ability in barley roots may be linked to WL stress tolerance (Zeng *et al.*, 2014). This suggestion is now fully validated by screening more barley cultivars differing in WL tolerance. We showed that the overall plant performance under waterlogged conditions (Fig. 1) correlated with whole-plant K^+^ tissue content (Fig. 3D), and that the inability of roots to retain K^+^ resulted in a loss of cell viability in sensitive genotypes (Figs. 2, 3). Moreover, the cell viability loss was more severe in the elongation zone tissues which also showed a significantly higher K^+^ loss as compared with mature root tissues (Fig. 4). In this context, the current results are consistent with previous studies which reported a reduction in K^+^ uptake in response to waterlogged conditions at the whole-plant level (Colmer and Greenway, 2010; Elzenga and van Veen, 2010; Zeng *et al.*, 2014), and exogenously applied K^+^ showed a beneficial effect on plant performance and alleviated the hostile effects of WL (Ashraf *et al.*, 2011; Wang *et al.*, 2013). Taken together, the data reported in the present study implicate cytosolic K^+^ retention as a key determinant of plant adaptive ability to hypoxia stress.

Interestingly, hypoxia-induced K^+^ efflux from the elongation zone increased in the first hours after stress onset but was then reduced to zero (Figs. 4A, 5A). These results may be indicative of the signalling role of K^+^ in this tissue. It was suggested that the transient decline in the cytosolic K^+^ pool may help plants to survive energy crises by ‘shutting down’ many energy-dependent processes such as protein synthesis and entering into the defence mode (Demidchik *et al.*, 2014; Shabala *et al.*, 2014). This concept, developed largely for salinity stress signalling (Shabala, 2017), appears to be valid for hypoxia signalling as well. Under this scenario, a quick loss of K^+^ from the cytosol and associated reduction in the enzymatic activity would serve as a push to redirect available energy from the energy-expensive processes of structural protein synthesis, to defence-related processes, such as the prevention of cytosolic acidification, detoxification of reactive oxygen species (ROS), and production of molecular chaperones (Shabala *et al.*, 2014; Zeng *et al.*, 2014). This approach may significantly reduce the consumption of ATP and thus increase the overall fraction of the available ATP pool to fuel the continued operation of H^+^-ATPase to sustain MP under O_2_-limited conditions. Once the signalling is over and expression of the appropriate genes has been triggered, K^+^ efflux from the elongation zone is reduced to zero (at 24 h and 48 h time points; Fig. 4A), to prevent further depletion of K^+^ resources and a loss of cell viability.

The kinetics of hypoxia-induced K^+^ fluxes across the PM in the mature zone are drastically different from those in the elongation zone. Here, a time-dependent progressive decline in K^+^ uptake is observed, with K^+^ uptake gradually turned into a net K^+^ loss in sensitive genotypes, as hypoxia progressed (Figs. 5A, 6). A strong positive correlation between the ability of mature zone cells to retain K^+^ and cell viability, and the overall WL stress tolerance of a specific genotype make it possible to recommend using steady-state K^+^ fluxes under these conditions (48 h hypoxia treatment; excised coleoptiles) as a physiological marker for breeding plants for WL stress tolerance. Our results suggested that the K^+^ flux measured in the mature root zone in both control and treated plants was uniform and sensitive enough to discriminate between tolerant and sensitive cultivars (Fig. 4B, C). Given that measurements are conducted in a steady state, each of them requires only 1.5–2 min, allowing ~30–40 specimens be measured in 1 h. Thus, the suggested protocol may be applied to screen a large number of double haploid populations for developing molecular markers and mapping quantitative trait loci (QTLs) for WL tolerance. It is worth noting that the root’s ability to retain K^+^ has never been targeted in the breeding programmes aimed at improving WL stress tolerance in any species.

### Regulation of the voltage-gated outward-rectifying K^+^ channels is critical for root K^+^ retention

K^+^ loss under hypoxic conditions may be triggered by a series of different potential factors such as (i) membrane depolarization and consequently channels opening; (ii) changes to the selectivity of K^+^ in non-selective membranes; and (iii) K^+^ leakage through ROS-stimulated channels (Shabala and Pottosin, 2014; Zeng *et al.*, 2014). Up to now, there are 75 genes of conjectural K^+^-permeable channels in the Arabidopsis genome which are enabling K^+^ transport across plant membranes. K^+^-selective channel genes are comprised of nine Shaker-type channels (Véry and Sentenac, 2002; Demidchik *et al.*, 2014;). Two of them, SKOR (located in the stellar tissue) and GORK (located in root epidermis), are classified as outward-rectifying K^+^ channels. SKOR facilitates K^+^ release from the xylem parenchyma cells to the xylem vessels, whereas GORK is playing its role in the leakage of K^+^ into external media (Demidchik *et al.*, 2014).

Earlier pharmacological studies suggested that hypoxia-induced changes in K^+^ fluxes could potentially be mediated by both voltage-dependent K^+^-inward (KIR) and K^+^-outward (KOR) rectifying channels (Pang *et al.*, 2006). However, strong depolarization of MP under hypoxic conditions reported in this work (Fig. 7) makes the involvement of KIR channels (such as AKT or KAT) thermodynamically impossible. This points to the GORK channel as the most likely candidate to mediate the observed effects of hypoxia in K^+^ transport in barley roots. Indeed, hypoxia-induced K^+^ loss and MP showed a strong association; less negative values of MP were accompanied by the severe loss of K^+^ in a very clear and genotype-specific manner (Figs. 4A, B, 7A, B). Also, the genotypes of tissues with more negative values of MP and less K^+^ loss showed better growth and cell viability when exposed to hypoxia or waterlogging (Figs. 1, 3A). Importantly, in addition to MP depolarization, GORK channels are also known to be activated by ROS (Demidchik *et al.*, 2010; García-Mata and Lamattina, 2010) which can be rapidly produced under hypoxia stress (Rhoads *et al.*, 2006).

The relative expression of *GORK* decreased in the mature zone; however, a 10-fold increase was found in the elongation zone when exposed to hypoxia (Fig. 8B), potentially explaining the higher sensitivity of this zone to hypoxia, as evidenced by viability staining (Fig. 2). Once again, this indicates that the GORK channel is the most likely candidate to control stress-induced K^+^ signalling and homeostasis in hypoxia-treated roots. The difference in the expression levels of *GORK* in the mature and elongation zone were mirrored by the changes of K^+^ effluxes when measured from different root zones of barley in hypoxic conditions (Figs. 4A, 5A). The present findings are in a full agreement with a recent report in which Arabidopsis showed a WL-tolerant phenotype after knocking out GORK channels (Wang *et al.*, 2016).

### H^+^-ATPase activity and/or expression determines GORK operation

Oxygen plays a key role in the process of efficient production of ATP in aerobic organisms (Voesenek *et al.*, 2006). Oxygen-deficient conditions lead towards energy crises by limiting O_2_ availability for ATP production, which results in a limited supply of energy to fuel H^+^-ATPase pumps which enable H^+^ extrusion (Bailey-Serres and Voesenek, 2008; Licausi and Perata, 2009; Shabala *et al.*, 2014). H^+^ pumps are the main electrogenic systems responsible for maintaining negative membrane potential values at the root PM (Teakle *et al.*, 2013; Shabala *et al.*, 2014). At the same time, they are also major consumers of ATP. In this study, the role of H^+^-ATPase as a key regulator of intracellular K^+^ homeostasis was shown directly in experiments by modifying O_2_ transportation to the shoot in experiments with excised coleoptiles (that operate as a snorkel under oxygen-limited conditions). The intact roots showed a significantly higher H^+^ efflux as compared with excised roots when measured from the mature root zone of barley under hypoxia stress (Figs. 4, 6). The barley cultivars showed a strong correlation between H^+^ flux and PM depolarization when measured from intact roots (Figs. 4, 7) which was further confirmed by the significant down-regulation of H^+^ -ATPase transcript levels. H^+^ efflux was strongly inhibited by vanadate (Fig. 7C, D), a known inhibitor of PM H^+^-ATPase. Our results showing reduction in H^+^-ATPase expression are consistent with previously reported results of a significant reduction in the H^+^-ATPase activity after only 2 h of anoxic treatment in pea epicotyls (Koizumi *et al.*, 2011).

Potentially, coleoptile excision may also interfere with the root K^+^ uptake and/or retention due to disrupted K^+^ cycling between shoots and roots. However, our early experiments showed that the effect of coleoptile excision on root K^+^ fluxes and MP was identical to that achieved by full coleoptile submergence (Zeng *et al.*, 2014) making the above scenario unlikely.

The other two categories of H^+^ pumps are the tonoplast-located vacuolar H^+^-ATPase (V-ATPase) and vacuolar H^+^-inorganic pyrophosphatase (V-PPase). These pumps show important roles for the accumulation of key ions in the vacuoles by generating an electrochemical H^+^ gradient through vacuolar membranes (Sze *et al.*, 1992). The V-ATPase pump plays a crucial role in avoiding cytosolic vacuolar acidification under oxygen-deficient conditions (Greenway and Gibbs, 2003; Koizumi *et al.*, 2011). Insufficient supply of O_2_ reduced the PM and V-H^+^-ATPase activities (Zhao *et al.* 2012). In the state of an inadequate V-ATPase activity, the V-PPase performs as an additional or alternative powerhouse of the tonoplast (Greenway and Gibbs, 2003). It was suggested that a shift from V-ATPase- to V-PPase-driven H^+^ extrusion is useful to roots with limited oxygen availability (Greenway and Gibbs, 2003). The breakdown of PPi is more favourable to ATP levels when ATP availability and cytoplasmic pH decrease due to hypoxia (Felle, 2005). In this study, the transcript levels of two V-PPase-coding genes, *HVP1* and *HVP10*, were regulated differently depending on the type of tissues. A substantial up-regulation of *HVP10* expression was measured from the elongation zone which was more sensitive to hypoxia as compared with the mature zone where it showed a reduction in the transcript level (Fig. 8D). In the mature zone, that under natural conditions has a better access to oxygen (Zeng *et al.*, 2014) and thus is capable of maintaining higher H^+^-ATPase activity (as judged by a more negative MP; Fig. 7), the up-regulation of V-PPase may not be required. The opposite scenario occurs in the elongation zone. In this context, our results are consistent with previous reports (Carystinos *et al.*, 1995; Harada *et al.*, 2007) of a massive increase in the transcript level of V-PPase after plant exposure to anoxic conditions followed by its decrease upon return to the well-aerated state. It should be noted that V-PPase activity shows a strong K^+^ dependence (Obermeyer *et al.*, 1996). Given such poor K^+^ retention ability in the elongation zone (Fig. 5), one can assume that the efficiency of V-PPase operation in this tissue would be reduced under hypoxic conditions. Thus, the observed increase in the transcript level of *HVP10* may be interpreted as a plant’s attempt to compensate for decreased V-PPase activity by increasing the number of functional units, to protect this zone from cytosolic acidosis under hypoxia stress.

## Supplementary data

Supplementary data are available at *JXB* online.

Fig. S1. Different steps of the experimental procedures.

Fig. S2. Correlation between K^+^ content in the shoot under waterlogging stress and net K^+^ flux in the mature root zone under hypoxia stress.

Supplementary Material

## References

[CIT0001] Alcántara E, de la Guardia MD, Romera FJ 1991 Plasmalemma redox activity and H^+^ extrusion in roots of fe-deficient cucumber plants. Plant Physiology 96, 1034–1037. 16668294 10.1104/pp.96.4.1034 PMC1080889

[CIT0002] Anschütz U, Becker D, Shabala S 2014 Going beyond nutrition: regulation of potassium homoeostasis as a common denominator of plant adaptive responses to environment. Journal of Plant Physiology 171, 670–687. 24635902 10.1016/j.jplph.2014.01.009

[CIT0003] Arnell NW, Liv C 2001 Hydrology and water resources. In: McCarthy JJ, Canziani OF, Leary NA, Dokken DJ, White KS, eds. Climate change: impacts, adaptation and vulnerability. Cambridge: Cambridge University Press, 191–233.

[CIT0004] Ashraf MA, Ahmad MSA, Ashraf M, Al-Qurainy F, Ashraf MY 2011 Alleviation of waterlogging stress in upland cotton (*Gossypium hirsutum* L.) by exogenous application of potassium in soil and as a foliar spray. Crop and Pasture Science 62, 25–38.

[CIT0005] Ashraf M, Rehman H 1999 Mineral nutrient status of corn in relation to nitrate and long-term waterlogging. Journal of Plant Nutrition 22, 1253–1268.

[CIT0006] Bailey-Serres J, Colmer TD 2014 Plant tolerance of flooding stress—recent advances. Plant, Cell and Environment 37, 2211–2215. 10.1111/pce.12420 25074340

[CIT0007] Bailey-Serres J, Voesenek LA 2008 Flooding stress: acclimations and genetic diversity. Annual Review of Plant Biology 59, 313–339. 10.1146/annurev.arplant.59.032607.092752 18444902

[CIT0008] Becker D, Hoth S, Ache P, Wenkel S, Roelfsema MR, Meyerhoff O, Hartung W, Hedrich R 2003 Regulation of the ABA-sensitive Arabidopsis potassium channel gene GORK in response to water stress. FEBS Letters 554, 119–126. 14596925 10.1016/s0014-5793(03)01118-9

[CIT0009] Board J 2008 Waterlogging effects on plant nutrient concentrations in soybean. Journal of Plant Nutrition 31, 828–838.

[CIT0010] Bose J, Babourina O, Shabala S, Rengel Z 2010 Aluminium-induced ion transport in Arabidopsis: the relationship between Al tolerance and root ion flux. Journal of Experimental Botany 61, 3163–3175. 20497972 10.1093/jxb/erq143 PMC2892157

[CIT0011] Buwalda F, Thomson C, Steigner W, Barrett-Lennard E, Gibbs J, Greenway H 1988 Hypoxia induces membrane depolarization and potassium loss from wheat roots but does not increase their permeability to sorbitol. Journal of Experimental Botany 39, 1169–1183.

[CIT0012] Carystinos GD, MacDonald HR, Monroy AF, Dhindsa RS, Poole RJ 1995 Vacuolar H(+)-translocating pyrophosphatase is induced by anoxia or chilling in seedlings of rice. Plant Physiology 108, 641–649. 7610161 10.1104/pp.108.2.641 PMC157384

[CIT0013] Chen Z, Newman I, Zhou M, Mendham N, Zhang G, Shabala S 2005 Screening plants for salt tolerance by measuring K^+^ flux: a case study for barley. Plant, Cell and Environment 28, 1230–1246.

[CIT0014] Close D, Davidson N 2003 Long-term waterlogging: nutrient, gas exchange, photochemical and pigment characteristics of *Eucalyptus nitens* saplings. Russian Journal of Plant Physiology 50, 843–847.

[CIT0015] Colmer TD, Greenway H 2011 Ion transport in seminal and adventitious roots of cereals during O_2_ deficiency. Journal of Experimental Botany 62, 39–57. 20847100 10.1093/jxb/erq271

[CIT0016] Colmer T, Munns R, Flowers T 2006 Improving salt tolerance of wheat and barley: future prospects. Australian Journal of Experimental Agriculture 45, 1425–1443.

[CIT0017] Cuin TA, Betts SA, Chalmandrier R, Shabala S 2008 A root’s ability to retain K^+^ correlates with salt tolerance in wheat. Journal of Experimental Botany 59, 2697–2706. 18495637 10.1093/jxb/ern128 PMC2486465

[CIT0018] Cuin TA, Shabala S 2005 Exogenously supplied compatible solutes rapidly ameliorate NaCl-induced potassium efflux from barley roots. Plant and Cell Physiology 46, 1924–1933. 16223738 10.1093/pcp/pci205

[CIT0019] Demidchik V, Cuin TA, Svistunenko D, Smith SJ, Miller AJ, Shabala S, Sokolik A, Yurin V 2010 Arabidopsis root K^+^-efflux conductance activated by hydroxyl radicals: single-channel properties, genetic basis and involvement in stress-induced cell death. Journal of Cell Science 123, 1468–1479. 20375061 10.1242/jcs.064352

[CIT0020] Demidchik V, Straltsova D, Medvedev SS, Pozhvanov GA, Sokolik A, Yurin V 2014 Stress-induced electrolyte leakage: the role of K^+^-permeable channels and involvement in programmed cell death and metabolic adjustment. Journal of Experimental Botany 65, 1259–1270. 24520019 10.1093/jxb/eru004

[CIT0021] Dobrovičová S, Dobrovič R, Dobrovič J 2015 The economic impact of floods and their importance in different regions of the world with emphasis on Europe. Procedia Economics and Finance 34, 649–655.

[CIT0022] Dreyer I, Uozumi N 2011 Potassium channels in plant cells. FEBS Journal 278, 4293–4303. 21955642 10.1111/j.1742-4658.2011.08371.x

[CIT0023] Elzenga JTM, van Veen H 2010 Waterlogging and plant nutrient uptake. In: Mancuso S, Shabala S, eds. Waterlogging signalling and tolerance in plants. Berlin: Springer-Verlag, 23–35.

[CIT0024] Felle HH 2005 pH regulation in anoxic plants. Annals of Botany 96, 519–532. 16024558 10.1093/aob/mci207 PMC4247022

[CIT0025] Gambale F, Uozumi N 2006 Properties of shaker-type potassium channels in higher plants. Journal of Membrane Biology 210, 1–19. 16794778 10.1007/s00232-006-0856-x

[CIT0026] García-Mata C, Lamattina L 2010 Hydrogen sulphide, a novel gasotransmitter involved in guard cell signalling. New Phytologist 188, 977–984. 20831717 10.1111/j.1469-8137.2010.03465.x

[CIT0027] Gibbs J, Greenway H 2003 Review: mechanisms of anoxia tolerance in plants. I. Growth, survival and anaerobic catabolism. Functional Plant Biology 30, 353. 10.1071/PP98095_ER 32689018

[CIT0028] Greenway H, Gibbs J 2003 Review: mechanisms of anoxia tolerance in plants. II. Energy requirements for maintenance and energy distribution to essential processes. Functional Plant Biology 30, 999–1036. 10.1071/PP98096 32689085

[CIT0029] Harada T, Satoh S, Yoshioka T, Ishizawa K 2007 Anoxia-enhanced expression of genes isolated by suppression subtractive hybridization from pondweed (*Potamogeton distinctus* A. Benn.) turions. Planta 226, 1041–1052. 17503072 10.1007/s00425-007-0537-8

[CIT0030] Heuer B 2003 Influence of exogenous application of proline and glycinebetaine on growth of salt-stressed tomato plants. Plant Science 165, 693–699.

[CIT0031] Hirabayashi Y, Mahendran R, Koirala S, Konoshima L, Yamazaki D, Watanabe S, Kim H, Kanae S 2013 Global flood risk under climate change. Nature Climate Change 3, 816–821.

[CIT0032] Hoagland DR, Arnon DI 1950 The water-culture method for growing plants without soil. California Agricultural Experiment Station, Circular-347.

[CIT0033] Ismail AM, Heuer S, Thomson MJ, Wissuwa M 2007 Genetic and genomic approaches to develop rice germplasm for problem soils. Plant Molecular Biology 65, 547–570. 17703278 10.1007/s11103-007-9215-2

[CIT0034] Jayakannan M, Bose J, Babourina O, Rengel Z, Shabala S 2013 Salicylic acid improves salinity tolerance in Arabidopsis by restoring membrane potential and preventing salt-induced K^+^ loss via a GORK channel. Journal of Experimental Botany 64, 2255–2268. 23580750 10.1093/jxb/ert085 PMC3654417

[CIT0035] Koizumi Y, Hara Y, Yazaki Y, Sakano K, Ishizawa K 2011 Involvement of plasma membrane H^+^-ATPase in anoxic elongation of stems in pondweed (*Potamogeton distinctus*) turions. New Phytologist 190, 421–430. 21232059 10.1111/j.1469-8137.2010.03605.x

[CIT0036] Koyama H, Toda T, Yokota S, Dawair Z, Hara T 1995 Effects of aluminum and pH on root growth and cell viability in *Arabidopsis thaliana* strain Landsberg in hydroponic culture. Plant and Cell Physiology 36, 201–205.

[CIT0037] Kreuzwieser J, Gessler A 2010 Global climate change and tree nutrition: influence of water availability. Tree Physiology 30, 1221–1234. 20581013 10.1093/treephys/tpq055

[CIT0038] Kronzucker HJ, Szczerba MW, Schulze LM, Britto DT 2008 Non-reciprocal interactions between K^+^ and Na^+^ ions in barley (*Hordeum vulgare* L.). Journal of Experimental Botany 59, 2793–2801. 18562445 10.1093/jxb/ern139 PMC2486474

[CIT0039] Licausi F, Perata P 2009 Low oxygen signaling and tolerance in plants. Advances in Botanical Research 50, 139–198.

[CIT0040] Maathuis FJ 2009 Physiological functions of mineral macronutrients. Current Opinion in Plant Biology 12, 250–258. 19473870 10.1016/j.pbi.2009.04.003

[CIT0041] Malik AI, English JP, Colmer TD 2009 Tolerance of *Hordeum marinum* accessions to O_2_ deficiency, salinity and these stresses combined. Annals of Botany 103, 237–248. 18701600 10.1093/aob/mcn142 PMC2707305

[CIT0042] Mugnai D 2011 The Schrödinger–Poisson system with positive potential. Communications in Partial Differential Equations 36, 1099–1117.

[CIT0043] Newman IA 2001 Ion transport in roots: measurement of fluxes using ion-selective microelectrodes to characterize transporter function. Plant, Cell and Environment 24, 1–14. 10.1046/j.1365-3040.2001.00661.x 11762438

[CIT0044] Obermeyer G, Sommer A, Bentrup FW 1996 Potassium and voltage dependence of the inorganic pyrophosphatase of intact vacuoles from *Chenopodium rubrum*. Biochimica et Biophysica Acta 1284, 203–212. 8914585 10.1016/s0005-2736(96)00130-7

[CIT0045] Pang JY, Newman I, Mendham N, Zhou M, Shabala S 2006 Microelectrode ion and O_2_ fluxes measurements reveal differential sensitivity of barley root tissues to hypoxia. Plant, Cell and Environment 29, 1107–1121. 10.1111/j.1365-3040.2005.01486.x 17080937

[CIT0046] Pang J, Zhou M, Mendham N, Shabala S 2004 Growth and physiological responses of six barley genotypes to waterlogging and subsequent recovery. Crop and Pasture Science 55, 895–906.

[CIT0047] Pezeshki S, DeLaune R, Anderson P 1999 Effect of flooding on elemental uptake and biomass allocation in seedlings of three bottomland tree species. Journal of Plant Nutrition 22, 1481–1494.

[CIT0048] Ponnamperuma F 1984 Effects of flooding on soils. In: Kozlowski TN, ed. Flooding and plant growth. New York: Academic Press, 9–45.

[CIT0049] Rhoads DM, Umbach AL, Subbaiah CC, Siedow JN 2006 Mitochondrial reactive oxygen species. Contribution to oxidative stress and interorganellar signaling. Plant Physiology 141, 357–366. 16760488 10.1104/pp.106.079129 PMC1475474

[CIT0050] Seneviratne SI, Nicholls N, Easterling D, Goodess CM, Kanae S, Kossin J, Luo Y, Marengo J, McInnes K, Rahimi M 2012 Changes in climate extremes and their impacts on the natural physical environment. In: Field CB, Barros V, Stocker TF, et al, eds. Managing the risks of extreme events and disasters to advance climate change adaptation. Cambridge: Cambrige University Press, 109–230.

[CIT0051] Setter T, Waters I 2003 Review of prospects for germplasm improvement for waterlogging tolerance in wheat, barley and oats. Plant and Soil 253, 1–34.

[CIT0052] Shabala S 2009 Salinity and programmed cell death: unravelling mechanisms for ion specific signalling. Journal of Experimental Botany 60, 709–712. 19269993 10.1093/jxb/erp013

[CIT0053] Shabala S 2017 Signalling by potassium: another second messenger to add to the list?Journal of Experimental Botany 68, 4003–4007. 28922770 10.1093/jxb/erx238 PMC5853517

[CIT0054] Shabala S, Bose J, Fuglsang AT, Pottosin I 2016 On a quest for stress tolerance genes: membrane transporters in sensing and adapting to hostile soils. Journal of Experimental Botany 67, 1015–1031. 26507891 10.1093/jxb/erv465

[CIT0055] Shabala S, Cuin TA 2008 Potassium transport and plant salt tolerance. Physiologia Plantarum 133, 651–669. 18724408 10.1111/j.1399-3054.2007.01008.x

[CIT0056] Shabala S, Pottosin I 2014 Regulation of potassium transport in plants under hostile conditions: implications for abiotic and biotic stress tolerance. Physiologia Plantarum 151, 257–279. 24506225 10.1111/ppl.12165

[CIT0057] Shabala S, Shabala L, Barcelo J, Poschenrieder C 2014 Membrane transporters mediating root signalling and adaptive responses to oxygen deprivation and soil flooding. Plant, Cell and Environment 37, 2216–2233. 10.1111/pce.12339 24689809

[CIT0058] Shabala S, Shabala L, Gradmann D, Chen Z, Newman I, Mancuso S 2006 Oscillations in plant membrane transport: model predictions, experimental validation, and physiological implications. Journal of Experimental Botany 57, 171–184. 16330526 10.1093/jxb/erj022

[CIT0059] Shabala S, Shabala S, Cuin TA, Pang J, Percey W, Chen Z, Conn S, Eing C, Wegner LH 2010 Xylem ionic relations and salinity tolerance in barley. The Plant Journal 61, 839–853. 20015063 10.1111/j.1365-313X.2009.04110.x

[CIT0060] Smethurst CF, Garnett T, Shabala S 2005 Nutritional and chlorophyll fluorescence responses of lucerne (*Medicago sativa*) to waterlogging and subsequent recovery. Plant and Soil 270, 31–45.

[CIT0061] Smethurst CF, Rix K, Garnett T, Auricht G, Bayart A, Lane P, Wilson SJ, Shabala S 2008 Multiple traits associated with salt tolerance in lucerne: revealing the underlying cellular mechanisms. Functional Plant Biology 35, 640–650. 10.1071/FP08030 32688819

[CIT0062] Sondergaard TE, Schulz A, Palmgren MG 2004 Energization of transport processes in plants. roles of the plasma membrane H^+^-ATPase. Plant Physiology 136, 2475–2482. 15375204 10.1104/pp.104.048231 PMC523315

[CIT0063] Sun J, Dai S, Wang R, et al 2009 Calcium mediates root K^+^/Na^+^ homeostasis in poplar species differing in salt tolerance. Tree Physiology 29, 1175–1186. 19638360 10.1093/treephys/tpp048

[CIT0064] Sze H, Ward JM, Lai S, Perera I 1992 Vacuolar-type H^+^-translocating ATPases in plant endomembranes: subunit organization and multigene families. Journal of Experimental Biology 172, 123–135. 9874730 10.1242/jeb.172.1.123

[CIT0065] Teakle NL, Amtmann A, Real D, Colmer TD 2010 *Lotus tenuis* tolerates combined salinity and waterlogging: maintaining O_2_ transport to roots and expression of an NHX1-like gene contribute to regulation of Na^+^ transport. Physiologia Plantarum 139, 358–374. 20444189 10.1111/j.1399-3054.2010.01373.x

[CIT0066] Teakle N, Bazihizina N, Shabala S, Colmer T, Barrett-Lennard E, Rodrigo-Moreno A, Läuchli A 2013 Differential tolerance to combined salinity and O_2_ deficiency in the halophytic grasses *Puccinellia ciliata* and *Thinopyrum ponticum*: the importance of K^+^ retention in roots. Environmental and Experimental Botany 87, 69–78.

[CIT0067] Trought M, Drew M 1980 The development of waterlogging damage in wheat seedlings (*Triticum aestivum* L.). Plant and Soil 54, 77–94.

[CIT0068] Turner F, Patrick W 1968 Chemical changes in waterlogged soils as a result of oxygen depletion. International Society of Soil Science Transactions 4, 53–56.

[CIT0069] Véry AA, Sentenac H 2002 Cation channels in the Arabidopsis plasma membrane. Trends in Plant Science 7, 168–175. 11950613 10.1016/s1360-1385(02)02262-8

[CIT0070] Voesenek LA, Colmer TD, Pierik R, Millenaar FF, Peeters AJ 2006 How plants cope with complete submergence. New Phytologist 170, 213–226. 16608449 10.1111/j.1469-8137.2006.01692.x

[CIT0071] Wang F, Chen Z-H, Liu X, Colmer TD, Shabala L, Salih A, Zhou M, Shabala S 2016 Revealing the roles of GORK channels and NADPH oxidase in acclimation to hypoxia in Arabidopsis. Journal of Experimental Botany 68, 3191–3204. 10.1093/jxb/erw378 PMC5853854 28338729

[CIT0072] Wang M, Zheng Q, Shen Q, Guo S 2013 The critical role of potassium in plant stress response. International Journal of Molecular Sciences 14, 7370–7390. 23549270 10.3390/ijms14047370 PMC3645691

[CIT0073] Ward JM, Mäser P, Schroeder JI 2009 Plant ion channels: gene families, physiology, and functional genomics analyses. Annual Review of Physiology 71, 59–82. 10.1146/annurev.physiol.010908.163204 PMC4790454 18842100

[CIT0074] Zeng F, Konnerup D, Shabala L, Zhou M, Colmer TD, Zhang G, Shabala S 2014 Linking oxygen availability with membrane potential maintenance and K^+^ retention of barley roots: implications for waterlogging stress tolerance. Plant, Cell and Environment 37, 2325–2338. 10.1111/pce.12422 25132404

[CIT0075] Zhao X, Li T, Sun Z 2012 Effects of prolonged root-zone CO_2_ treatment on morphological parameter and nutrient uptake of tomato grown in aeroponic system. Journal of Applied Botany and Food Quality 83, 212–216.

[CIT0076] Zhou M, Johnson P, Zhou G, Li C, Lance R 2012 Quantitative trait loci for waterlogging tolerance in a barley cross of Franklin×YuYaoXiangTian Erleng and the relationship between waterlogging and salinity tolerance. Crop Science 52, 2082–2088.

