# Supplementary material for: The ability to regulate voltage-gated K+-permeable channels in the mature root epidermis is essential for waterlogging tolerance in barley
Source: J Exp Bot. 2017 Dec 28;69(3):667–80. doi: 10.1093/jxb/erx429 (PMC5853535; doi:10.1093/jxb/erx429)
Supplement: Supplementary Material [file erx429_suppl_supplementary_material.pdf]

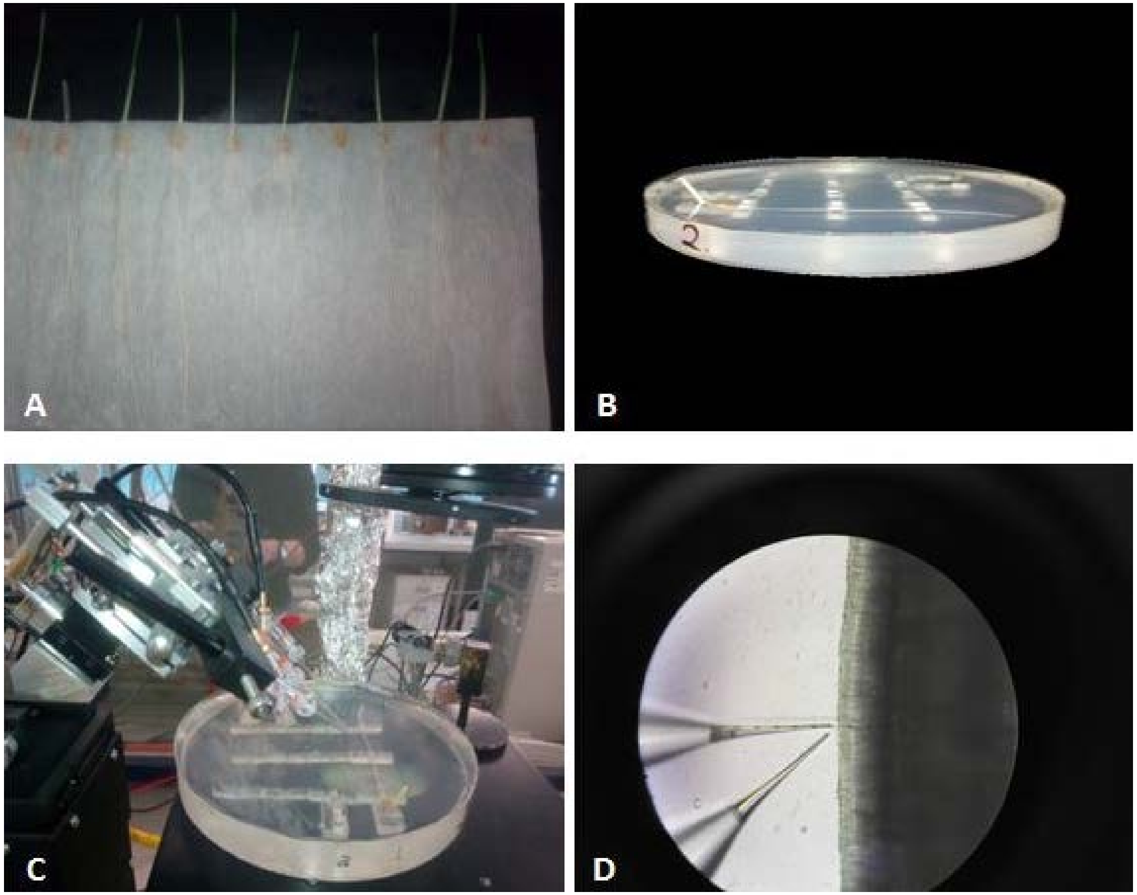

Fig. S1 Different steps of experimental procedures. (A) Plants are grown in paper rolls. (B) Plants are fixed in a chamber and treated with 0.2% agar for different timings. (C) Plant seedling is transferred to Faraday cage. (D) Ion selective electrodes are positioned for the measurement from the root epidermis.

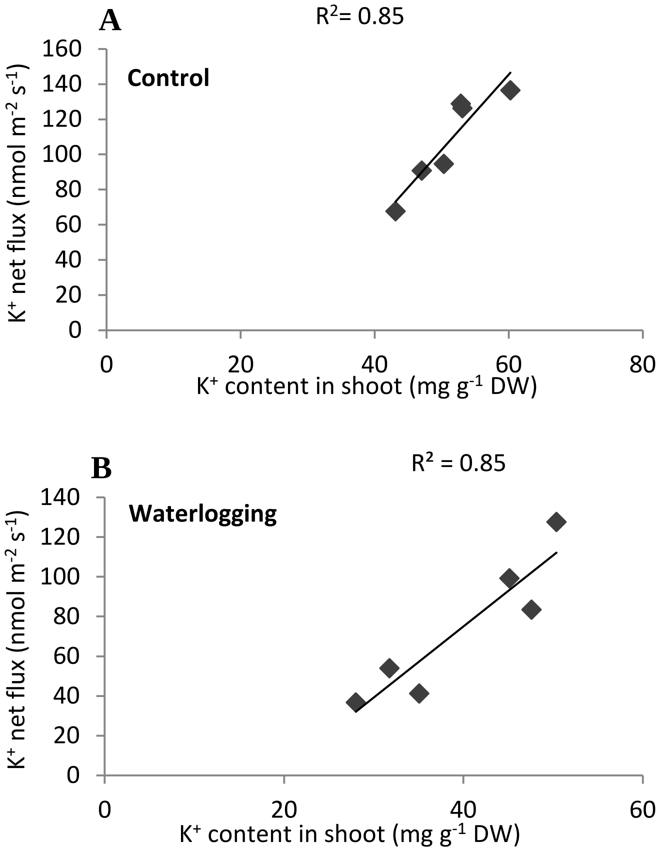

Fig. S2 Correlation between K^+ content in the shoot (mg g^{-1} DW) under waterlogging stress and net K^+ flux (nmol $m^{-2}s^{-1}$) in the mature root zone under hypoxia stress. Each point represents the mean value for one of six cultivars mentioned in the main text.

Supplementary Table S1: Primers used in the gene expression analysis

Gene name	Forward primer	Reverse primer
HvGAPDH	5’-GTGAGGCTGGTGCTGATTACG-3’	5’-TGGTGCAGCTAGCATTTGAGAC-3’
HvPMHA	5’-GCTGGTGTTATCTGGCTCTTC-3’	5’-CTCTTCTCTTGGCTT GCTCAG-3’
HvGORK	5’-CCACACGAGGCGAAGAAG-3’	5’-GAGGAATCCACAGCATCACC-3’
HVP1	5’-GAAGACTGTGCATAGCTGGC-3’	5’-ACATTGGTAGCAGCTCCAGT-3’
HVP10	5’-AGATGACCCAAGGAACCCAG-3’	5’-GCAAAGAGTGTGGTGAGCAA-3’
